# A Case of Chyloperitoneum Secondary to Follicular Lymphoma and a Review of Prognostic Implications

**DOI:** 10.1155/2016/4625819

**Published:** 2016-06-26

**Authors:** Megan Jagosky, Brice Taylor, Stephanie Parks Taylor

**Affiliations:** Carolinas Medical Center, Medical Education Building, 5th floor, P.O. Box 32861, Charlotte, NC 28232-2861, USA

## Abstract

Chyloperitoneum, or chylous ascites, is a rare condition characterized by milky-appearing fluid with elevated triglyceride content and the presence of chylomicrons. Malignancy, specifically lymphoma, is reported to be the predominant cause in Western countries. Previously, the prognosis for patients with chyloperitoneum due to lymphoma has been reported as poor. We present a case of chyloperitoneum and chylothorax due to follicular lymphoma with excellent response to bendamustine and Rituxan. A review of the literature indicates that patients with chyloperitoneum associated with lymphoma generally have a favorable response to contemporary treatment regimens.

## 1. Background

Chyloperitoneum, also known as chylous ascites, is a rare condition characterized by milky-appearing lymph fluid in the abdominal cavity. It presents slowly with gradual, painless abdominal distension. The patient may experience weight gain and dyspnea as the fluid exerts pressure on the diaphragm. Paracentesis is necessary for diagnosis as it will reveal turbid, milky fluid that is distinguished by chylomicrons and an elevated triglyceride content (>200 mg/dL). Abdominal malignancies, specifically lymphoma, is reported to be the predominant cause in Western countries [1]. The most likely mechanism of fluid accumulation is impingement of the drainage system and possible direct invasion into the lymphatics.

In 1982, Press et al. reviewed the largest series of patients with chyloperitoneum due to lymphoma and found prognosis to be poor—only 2 of 13 patients experienced long-term survival (greater than three months) [2]. One would predict that survival has improved since then with the emergence of superior chemotherapy regimens. We present a case of chylous ascites and chylothorax due to follicular lymphoma and review the literature with specific focus on response to treatment.

## 2. Case Description

A 78-year-old woman presented to an outside facility with dyspnea and abdominal fullness. Physical examination revealed abdominal distension with a fluid wave and decreased breath sounds with dullness to percussion in the left hemithorax. Paracentesis and thoracentesis were both performed, revealing creamy opaque fluid with an elevated triglyceride content. Cytological examination of the peritoneal fluid showed chylomicrons. Prior to presentation to our institution, she underwent thoracentesis four times and paracentesis five times with rapid recurrence of symptoms.

**Figure 1:**
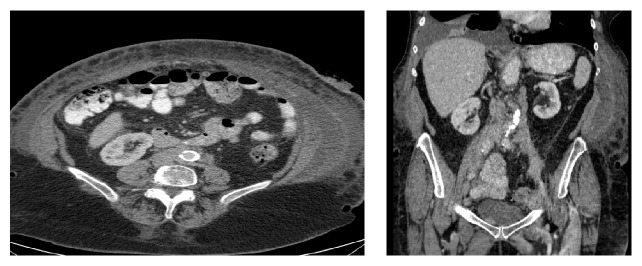
Coronal and transverse CT imaging of para-aortic lymphadenopathy.

**Figure 2:**
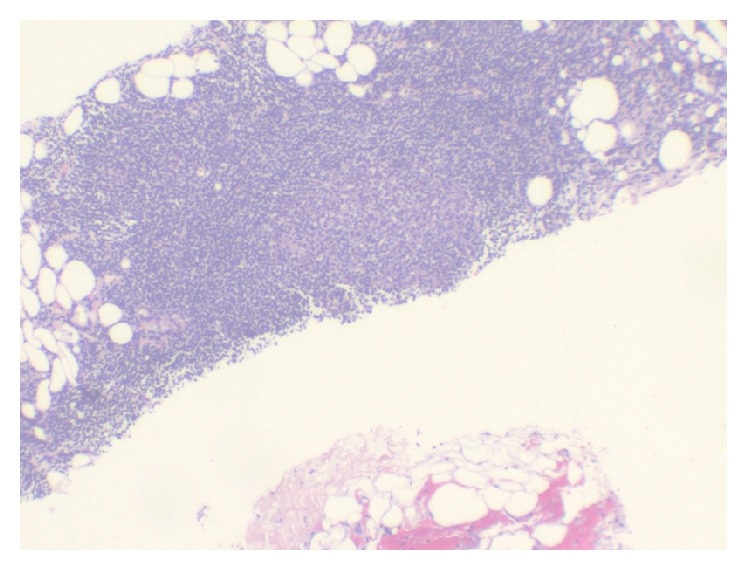
Lymph node biopsy pathology displaying small lymphoid cell proliferation.

On presentation to our institution, computed tomography (CT) scan of the abdomen and pelvis revealed para-aortic lymphadenopathy (Figure 1). A CT scan of the thorax showed a large left pleural effusion with scattered mediastinal lymph nodes, but they were not enlarged using the size criteria. A CT-guided lymph node biopsy of the para-aortic abdominal nodes revealed small lymphoid cell proliferation (Figure 2). Flow cytometry analysis of the sample lymph node biopsied demonstrated a monoclonal B-cell population positive for CD10, CD19, and CD20 and negative for CD3 and CD5. Ki-67, a protein marker for cell proliferation, was low within the neoplastic follicles (less than 10%).*‬‬‬*

Based on the histologic appearance and the low expression of Ki-67, the patient was diagnosed with low-grade follicular lymphoma. Due to the bulkiness of her disease with the multiple recurrent effusions, chemotherapy was pursued. Further staging workup with bone marrow biopsy and PET scan was deferred since it would not have changed management with the plan to pursue systemic therapy, regardless. She received four cycles of bendamustine and rituximab with complete resolution of both the ascites and pleural effusion. At one-year follow-up since complete resolution of her disease, she remained asymptomatic without reoccurrence of effusions; therefore, further chemotherapy was not pursued.

## 3. Discussion

Serous effusions are relatively common among malignant lymphomas. The most common effusion site is pleural, affecting 20–30% of NHL and Hodgkin lymphoma (HL), followed by pericardial and peritoneal [14]. Presence of an effusion historically portends a poor prognosis with higher risk for relapse after chemotherapy and decreased overall survival. While effusions in general are common in lymphoma, chylous effusions are exceedingly rare.

Targeting the underlying cause of chylous ascites is crucial to resolution, as in our patient who received follicular lymphoma-directed therapy. Treatment preferences for follicular lymphoma are varied since patients can live well for many years without treatment. Generally, stage I and stage II disease can be treated with watchful waiting or radiotherapy, whereas bulky stage II or stage III-IV can be treated with rituximab plus other biologic agents or chemotherapy, with or without radiotherapy [15]. Due to the rarity of follicular lymphoma-related chylous effusions, there are no guidelines for treatment. Conservative measures like dietary changes with protein rich, low-lipid foods can reduce chyle flow. Medium chain triglycerides are preferably consumed since they are directly absorbed and transported as free chain fatty acids and glycerol in the portal vein. Consumption of long chain fatty acids should be minimized since their metabolism leads to monoglycerides and free fatty acids that require transport by chylomicrons through the intestinal lymphatics [16]. Conservative measures are often ineffective in lymphoma; therefore, if lymphadenopathy is present, chemotherapy, or radiotherapy, or both can be pursued. In refractory cases, surgery can be utilized to ligate the thoracic duct or generate a pleuroperitoneal shunt [17].

To evaluate the current state of chyloperitoneum prognosis and treatment secondary to lymphoma, a search of the MED-LINE database was performed using the search terms “chylous ascites” or “chyloperitoneum” and “lymphoma”. Fourteen cases from 10 unique publications were found that described chylous ascites secondary to lymphoma [3–13] (Table 1). In addition to our patient, six cases had both chylous ascites and chylous pleural effusion, with two cases also having chylopericardium. As in our case, most were associated with low-grade lymphomas and had excellent response to a variety of different chemotherapy regimens. Of the 14 patients, three had HL and 11 had NHL. Of the NHL cases, four had diffuse large B-cell lymphoma (DLBCL), two had follicular lymphoma, two had small-cleaved cell (unspecified), one had Burkitt's lymphoma, one had T-cell lymphoma, and one was unspecified. The two patients with the more aggressive subtypes (T-cell and Burkitt's lymphoma) died from their disease.

Our review of the contemporary literature suggests that the advent of superior chemotherapy regimens has led to marked improvement in survival and resolution of symptoms in most patients. Prior to the development of these regimens, lymphoma-related chylous effusions carried a dismal prognosis with greater than 90% of patients dying within three months of diagnosis before 1982 [2]. In the majority of cases published since that time, chemotherapeutic regimens directed to specific lymphoma subtypes allowed for a significant beneficial effect. Twelve of the fourteen cases noted here had significant regression of disease and complete or near complete resolution of effusions. Long-term follow-up was only described in three cases with one patient surviving until 18 months [8] and another two surviving, symptom free, at least until the one-year and eight-month follow-up, respectively [10, 13]. Our patient is, to our knowledge, the first published case treated with bendamustine and rituximab. As in the other cases reviewed, her outcome was a positive one and, because of current therapies, she continued to thrive beyond one year after diagnosis.

## Figures and Tables

**Table 1:** Chyloperitoneum associated with lymphoma.

Case number	Author/year	Age/sex	Site	Diagnosis	Treatment	Outcome
1	Current case	78/F	Peritoneal, pleural,	Follicular lymphoma	Bendamustine + rituximab	Resolution
2	Arasawa, 2014 [3]	74/M	Peritoneal	DLBCL	R-CHOP	Resolution
3	Jiang, 2013 [4]	28/M	Peritoneal, pleural	Non-Hodgkin Lymphoma	DOLP + HDAra-C	Resolution
4	Yamamoto, 2013 [5]	87/M	Peritoneal	DLBCL	R-CHOP	Resolution
5	Etonyeaku, 2012 [6]	18/F	Peritoneal, pleural	Burkitt's lymphoma	MEV	Died due to treatment effects
6	Kashyap, 2011 [7]	21/F	Peritoneal, pleural, pericardial	DLBCL	R-CHOP	Resolution
7	Ionnidou-Papagiannaki 2009 [8]	38/M	Peritoneal, pleural, pericardial	T-cell lymphoma	CHOEP	Died due to GI bleeding
8	Gonen, 2007 [9]	53/M	Peritoneal	Hodgkin lymphoma	ABVD	Regression after two cycles chemotherapy
9	Ward, 2008 [10]	Middle age/F	Peritoneal	Follicular lymphoma	Chemotherapy unspecified	Resolution
10	Bachmeyer, 2004 [11]	71/M	Peritoneal	Follicular lymphoma	CHVP + interferon alpha	50% reduction
11	Oosterbosch, 1995 [12]	68/F	Peritoneal, pleural	DLBCL	CVP	Regression
12	Hufford, 1988 [13]	49/F	Peritoneal	Mixed cellularity (Hodgkin lymphoma)	CVP, doxorubicin followed by CHOP	Resolution
13	Hufford. 1988 [13]	67/F	Peritoneal	Small cleaved cell (NHL)	CVP	No response to chemotherapy, resolution after diuretics
14	Hufford, 1988 [13]	58/M	Peritoneal	Hodgkin lymphoma	MOPP	Resolution
15	Hufford, 1988 [13]	60/M	Peritoneal	Small cleaved cell (NHL)	CHOMP + bleomycin	Resolution

R-CHOP: rituximab, cyclophosphamide, doxorubicin, vincristine, and prednisone; CVP: cyclophosphamide, vincristine, and prednisolone; CHOMP: cyclophosphamide, doxorubicin, vincristine, and prednisone + bleomycin; MOPP: Mustargen, Oncovin, procarbazine, and prednisone; CHVP: cyclophosphamide, doxorubicin, etoposide, and prednisolone; ABVD: doxorubicin, bleomycin, vinblastine, and dacarbazine; CHOEP: cyclophosphamide, hydroxydaunorubicin, Oncovin, etoposide, and prednisone; DOLP: daunorubicin, vincristine, L-asparaginase, and prednisone; HDAra-C: high dose cytarabine; MEV: methotrexate, cyclophosphamide, and vincristine.
